# Clinical Features and Contributing Factors of Excessive Daytime Sleepiness in Chinese Obstructive Sleep Apnea Patients: The Role of Comorbid Symptoms and Polysomnographic Variables

**DOI:** 10.1155/2019/5476372

**Published:** 2019-07-10

**Authors:** Chuan Shao, Huan Qi, Ruyi Lang, Biyun Yu, Yaodong Tang, Lina Zhang, Xun Wang, Ling Wang

**Affiliations:** ^1^Department of Special Procurement Ward, The First Affiliated Hospital of Soochow University, Suzhou 215006, China; ^2^Department of Respiratory Medicine, Ningbo Medical Center Lihuili Eastern Hospital, Ningbo 315040, China; ^3^Department of Respiratory Medicine, Taipei Medical University Ningbo Medical Center, Ningbo 315040, China; ^4^Department of Preventive Medicine, School of Medicine, Ningbo University, Ningbo 315211, China; ^5^Department of Respiratory and Critical Care Medicine, Wuxi No. 2 People's Hospital, Nanjing Medical University, Wuxi 214002, China

## Abstract

**Background:**

The occurrence and severity of excessive daytime sleepiness (EDS) vary considerably among obstructive sleep apnea (OSA) patients. This study was designed to investigate the characteristics of EDS and identify its contributing factors in OSA patients.

**Methods:**

This was a cross-sectional study from a tertiary medical center in China. A total of 874 consecutive patients with newly diagnosed OSA were included. Subjective daytime sleepiness was assessed with the Epworth Sleepiness Scale (ESS). The subjects were assigned to the non-EDS group (582 patients), mild to moderate EDS group (227 patients), and severe EDS group (65 patients) according to the ESS scores. The clinical features and polysomnographic parameters were acquired and analyzed to identify the differences between groups and the determinants of EDS.

**Results:**

The age of patients with severe EDS (49.5 ± 11.3) was slightly greater than that of patients with mild to moderate EDS (44.5 ± 10.2) (*p* < 0.05) and non-EDS patients (45.2 ± 12.0) (*p* < 0.05). Body mass index (BMI) was highest in the severe EDS group (29.1 ± 3.6 kg/m^2^) (*p* < 0.0001), intermediate in the mild to moderate EDS group (27.9 ± 3.3 kg/m^2^), and lower in the non-EDS group (26.8 ± 3.3 kg/m^2^). Logistic regression analysis showed waist circumference, memory loss, work/commute disturbances, and sleep efficiency were independently associated with mild to moderate EDS, and the microarousal index, apnea-hypopnea index (AHI), and saturation impair time below 90% were independent contributing factors of mild to moderate EDS. Meanwhile, age, neck circumference, gasping/choking, memory loss, work/commute disturbances, and sleep latency were independently associated with severe EDS, and the AHI and mean SpO_2_ were independent contributing factors of severe EDS.

**Conclusions:**

OSA patients with various severities of EDS are more obese and have more comorbid symptoms compared to patients without EDS. Sleep fragmentation, respiratory events, and nocturnal hypoxia may be predictors of EDS. Comprehensive consideration of demographic, clinical, and polysomnographic factors is required when evaluating OSA patients.

## 1. Introduction

Obstructive sleep apnea (OSA) is the most common sleep disorder and is a chronic systemic condition. According to the latest epidemiological results of the HypnoLaus study, the prevalence of moderate to severe sleep-disordered breathing (≥15 events per h) in the general population was 23.4% in women and 49.7% in men [1]. With population aging and obesity increasing, the prevalence of OSA may increase further. OSA is characterized by recurrent upper airway obstruction/collapse, chronic intermittent hypoxia, and sleep fragmentation at night [2]. Untreated OSA is associated with many adverse clinical outcomes, including cardiovascular diseases, metabolic disorders, cognitive impairment, and malignancy [2]. Symptoms of OSA include daytime sleepiness, arousal during night sleep, morning headache, dry mouth, increased nocturia, memory loss, and irritability [3]. Symptoms can vary greatly from patient to patient.

Excessive daytime sleepiness (EDS) is a major symptom of OSA. Our previous study showed that 58.3% of OSA patients referred to a tertiary hospital had varying degrees of daytime sleepiness [4]. Daytime sleepiness is an important cause of traffic accidents and occupational incidents [5, 6] and is associated with cognitive impairment [7]. EDS severely affects quality of life and increases mortality [8, 9]. However, many OSA patients do not have EDS [10], and the reasons why some patients have EDS while some do not are important and warrant investigation. Although this issue is sometimes ignored, it has important clinical and epidemiological implications. OSA is currently considered a complex and heterogeneous condition, with varying symptoms, pathogenesis, comorbidities, and clinical outcomes from patient to patient [11]. Different phenotypes have been increasingly identified in OSA and may promote precise medicine. Daytime sleepiness has become a primary criterion for symptom-based OSA classification [12]. In-depth studies of the features and differences of OSA patients with or without EDS may help improve the screening, diagnosis, and prognosis of OSA in the future. For example, it is confirmed that continuous positive airway pressure (CPAP) can mitigate the risk factors of hypertension, insulin resistance, cardiovascular diseases, and endothelial dysfunction in OSA patients with EDS. However, studies have not reached the same conclusion for OSA patients without EDS [13]. The mechanisms of EDS in OSA patients are unknown. Researchers generally believe that, for OSA patients, recurrent apnea/hypopnea-induced frequent wake/sleep fragmentation and sleep structural disorders may cause EDS [14]. However, subsequent studies show that nocturnal hypoxia may be the major contributing factor for EDS [15]. However, some OSA patients still have daytime sleepiness following effective CPAP treatment to eliminate respiratory events [16]. Moreover, some studies have shown that the correlation between OSA severity and the Epworth Sleepiness Scale (ESS) score is weak or even nonexistent [17]. These data indicate that various factors may be involved in the pathogenesis of daytime sleepiness [18]. Research studies from animal models of OSA indicated that EDS was to some extent related to inflammation [13]. For example, CIH (chronic intermittent hypoxia) models that mimic the major pathophysiological characteristics of OSA had been shown to induce inflammation that may eventually promote EDS in certain ways [19, 20]. Besides, EDS may also be related to genetic predisposition [21]. Despite ongoing debate regarding the causes of EDS in OSA patients, comprehensive and in-depth studies are scarce, and prospective studies are lacking.

This study was designed to comprehensively investigate potential contributing factors of EDS in OSA patients based on clinical features and polysomnographic variables. The ESS is the most widely used clinical tool for evaluating subjective sleepiness [22]. In this study, OSA patients were assigned to the non-EDS group, mild to moderate EDS group, and severe EDS group based on ESS scores. Clinical features (including comorbid symptoms, important OSA-related signs, and comorbidities) and PSG variables (including sleep structure, respiratory events, and nocturnal hypoxia-related indicators) were analyzed to identify differences between the three groups and potential risk factors of EDS in OSA patients.

## 2. Methods

### 2.1. Study Population

**Figure 1:**
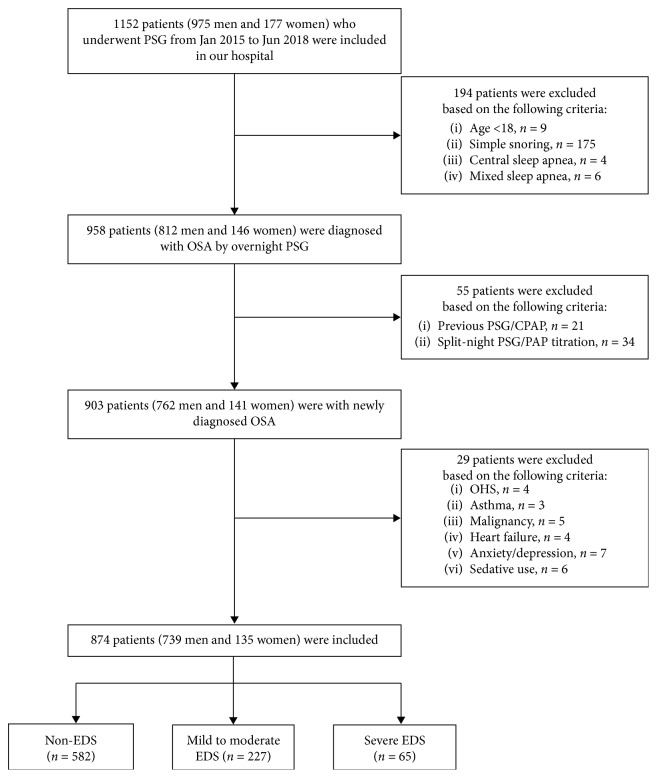
Flow chart of the patients' inclusion process. PSG: polysomnography; OSA: obstructive sleep apnea; CPAP: continuous positive airway pressure; PAP: positive airway pressure; OHS: obesity hypoventilation syndrome; EDS: excessive daytime sleepiness.

One thousand one hundred fifty-two patients who visited Ningbo Medical Center Lihuili Eastern Hospital and underwent overnight polysomnography (PSG) between January 2015 and June 2018 were screened consecutively. Eligible patients were newly diagnosed, treatment-naïve OSA patients. OSA was diagnosed according to the OSA diagnostic criteria from the International Classification of Sleep Disorders-Third Edition (ICSD-3) issued by the American Academy of Sleep Medicine (AASM) [23]. The exclusion criteria included the following: (1) previous OSA-associated treatment; (2) other sleep disorders, such as central sleep apnea; (3) obesity hypoventilation syndrome, bronchial asthma, malignant tumor, or congestive heart failure; and (4) mental illness or long-term use of sedative and hypnotic drugs. A total of 874 patients were enrolled in the study, including 739 men and 135 women with a mean age of 45.4 ± 11.6 years. The flow chart of the patients' inclusion process is shown in Figure 1. Each patient signed written informed consent before the start of the study, which was approved by the hospital's ethics committee.

### 2.2. Clinical Features

Two respiratory specialists collected a detailed medical history from each patient, performed OSA-related physical examinations, and recorded basic information and clinical features, including gender, age, height, weight, finger pulse oximetry (SpO_2_) during wakefulness before sleep, symptoms, and related medical history information, including comorbidities and other previous medical histories. Comorbidities were diagnosed based on relevant diagnostic criteria, test results, or a clear history of diagnosis and treatment. Body mass index (BMI) was calculated as weight (kg)/height^2^ (m). Neck circumference (NC) was measured at the level of the laryngeal prominence. Waist circumference (WC) was measured at the level between the lower edge of rib 12 and the upper edge of the iliac crest at the end of exhalation (before inhalation). The pharyngeal cavity and tonsils were examined. Mandibular retraction was diagnosed based on imaging studies. Pharyngeal stenosis and tonsil enlargement were diagnosed according to the Friedman classification system [24]. All the clinical features were collected during the day prior to the PSG recording.

### 2.3. EDS Assessment

On the day of admission, each patient independently completed the Chinese version of the ESS under the direction of a sleep specialist [25]. The ESS questionnaire consists of 8 short items, and each item is rated on a 0–3 scale (0: never doze off, 1: a low probability of dozing off, 2: a moderate probability of dozing off, and 3: a high probability of dozing off). Patients subjectively assessed the severity of daytime sleepiness under 8 different conditions: (1) sitting and reading, (2) watching TV, (3) sitting still in a public place (such as a theater or conference room), (4) taking a bus as a passenger for 1 hour, (5) sitting down and talking to someone, (6) sitting quietly after lunch without drinking, (7) sitting in the car, with the car stopping for a few minutes, and (8) lying down to rest in the afternoon (if applicable). The total score ranges from 0 to 24. An ESS score >10 indicates EDS, and an ESS score ≤10 indicates no EDS [26]. The 874 patients enrolled were divided into three groups based on ESS scores, including the non-EDS group (ESS ≤ 10) (*n*=582), mild to moderate EDS group (11 ≤ ESS ≤ 16) (*n*=227), and severe EDS group (ESS ≥ 17) (*n*=65).

### 2.4. Polysomnographic Study

The patients underwent overnight PSG in the hospital's sleep-monitoring room. On the day of PSG, the patients were instructed to refrain from alcohol, tea, coffee, and sedative and hypnotic drugs. An Alice 5 system (Respironics, Philips, Pittsburgh, Pennsylvania, USA) was used for overnight PSG, which started when the patients were ready to sleep at night and ended when the patients woke up the next morning (at least seven hours). During the PSG, data of electroencephalogram (EEG), electrooculogram (EOG), mandibular electromyogram (EMG), electrocardiogram (ECG), oronasal airflow (including thermal airflow signals and pressure airflow signals), chest and abdomen respiratory movement, and pulse oximetry measurements were recorded. Overnight PSG and manual scoring and interpretation of PSG results were completed by experienced sleep specialists and PSG technicians. Sleep stages, microarousals, and respiratory events were interpreted based on the AASM Manual for the Scoring of Sleep and Associated Events [27]. Sleep efficiency is calculated as the total sleep time (TST)/time in bed × 100%, and the percentage of each sleep stage is the time spent in this sleep stage as a percentage of TST. The microarousal index (MAI) is the mean frequency of microarousal per hour of sleep. Apnea is considered if the amplitude of oronasal thermal airflow is reduced by more than 90% from baseline for at least 10 seconds. Hypopnea is considered if the amplitude of oronasal pressure airflow is reduced by more than 30% from baseline, with a decrease in SpO_2_ by 3% or arousal for at least 10 seconds. The apnea-hypopnea index (AHI) is the mean frequency of apnea and hypopnea per hour of sleep. The oxygen desaturation index (ODI) is the mean frequency of SpO_2_ decreasing by ≥3% per hour of sleep. The SIT90 is the percentage of time with an SpO_2_ below 90% during TST. In addition, PSG variables include the time of wake after sleep onset (WASO), sleep latency (SL), the longest time of apnea (TAmax), the nadir SpO_2_, and the mean SpO_2_.

### 2.5. Statistical Analysis

Descriptive data were reported as mean values ± SD or percentage (%). Comparisons of continuous variables including demographic and PSG parameters across the three groups were performed using the one-way analysis of variance. Comparisons of categorical variables, including gender, symptoms, and comorbidities, were performed using the *χ* ^2^ test. Binary logistic regression was used for multivariate analysis. To identify possible contributors of EDS, two logistic regression analyses with backward selection comparing the mild or moderate EDS group versus the non-EDS group and the severe EDS group versus the non-EDS group were performed separately. All clinical and statistically significant variables were included as independent variables for logistic analysis. SPSS v22 (SPSS, Inc., Chicago, IL, USA) was used for statistical analysis, and *p* < 0.05 was considered statistically significant.

## 3. Results

### 3.1. Demographic Characteristics

The main demographics of the 874 OSA patients are shown in Table 1. No significant between-group differences were observed in gender (*p*=0.061) or awake SpO_2_ (*p*=0.097). The age of the severe EDS group (49.5 ± 11.3) was slightly greater than that of the mild to moderate EDS group (44.5 ± 10.2) (*p* < 0.05) and non-EDS group (45.2 ± 12.0) (*p* < 0.05). BMI was highest in the severe EDS group (29.1 ± 3.6) (*p* < 0.0001), intermediate in the mild to moderate EDS group (27.9 ± 3.3), and lower in the non-EDS group (26.8 ± 3.3). Both NC and WC of the severe EDS group (39.7 ± 3.4 and 102.1 ± 9.9) and mild to moderate EDS group (38.9 ± 3.2 and 101.0 ± 9.5) were greater than those of the non-EDS group (38.1 ± 4.1 and 96.1 ± 9.0) (all *p* < 0.05).

### 3.2. Clinical Manifestations and Comorbidities

Except for daytime sleepiness, results of other symptoms, physical examinations, comorbidities, and previous medical histories are shown in Table 2. In terms of comorbid symptoms, the prevalence of gasping/choking, dry mouth, memory loss, and irritability in the severe EDS group (58.5%, 75.4%, 81.5%, and 46.2%, respectively) (all *p* < 0.05) and mild to moderate EDS group (46.3%, 70.9%, 70.5%, and 43.6%, respectively) (all *p* < 0.05) was higher than that in the non-EDS group (33.7%, 61.9%, 55.3%, and 33.7%, respectively). The prevalence of morning headache, life disturbance, work disturbance, and commute disturbance was highest in the severe EDS group (36.9%, 61.5%, 64.6%, and 46.2%, respectively) (all *p* < 0.0001), intermediate in the mild or moderate EDS group (23.3%, 47.1%, 38.8%, and 26.4%, respectively), and lower in the non-EDS group (12.4%, 33%, 19.4%, and 7.6%, respectively). The prevalence of increased nocturia in the severe EDS group (47.7%) was higher than that in the mild to moderate EDS group (29.1%) (*p* < 0.05) and non-EDS group (25.4%) (*p* < 0.05). As to the comorbidities, the prevalence of type 2 diabetes mellitus (T2DM) in the severe EDS group (18.5%) (*p* < 0.05) and mild to moderate EDS group (12.3%) (*p* < 0.05) was higher than that in the non-EDS group (7%). The prevalence of other comorbidities was not statistically significant. Furthermore, the prevalence of tonsil enlargement in the mild to moderate EDS group (13.2%) was higher than that in the non-EDS group (7.2%) (*p* < 0.05), and the prevalence of pharyngeal stenosis in the severe EDS group (30.8%) (*p* < 0.05) and mild to moderate EDS group (22.5%) (*p* < 0.05) was higher than that in the non-EDS group (12.4%).

### 3.3. Polysomnographic Results

The results of the overnight polysomnographic study are shown in Table 3. The sleep efficiency, percentage of stage N1, MAI, AHI, TAmax, ODI, and SIT90 in the severe EDS group (88.2 ± 10.3%, 48.5 ± 19.5%, 51.4 ± 22.9 h^−1^, 55.7 ± 24.7 h^−1^, 66.1 ± 28.5 s, 57.2 ± 26.2 h^−1^, and 26.5 ± 23.5%, respectively) (all *p* < 0.05) and mild to moderate EDS group (87.3 ± 10.3%, 46.4 ± 22.6%, 51.5 ± 20.7 h^−1^, 54.4 ± 22.8 h^−1^, 68.7 ± 28.7 s, 54.8 ± 24.3 h^−1^, and 22.6 ± 32.4%, respectively) (all *p* < 0.05) were higher than those in the non-EDS group (83.5 ± 12.9%, 40.7 ± 17.3%, 40.8 ± 19.4 h^−1^, 36.6 ± 23.2 h^−1^, 56.2 ± 25.7 s, 36.9 ± 24.1 h^−1^, and 12.3 ± 16.6%, respectively). The WASO, REM (%), nadir SpO_2_, and mean SpO_2_ in the severe EDS group (34.8 ± 39.5 min, 6.6 ± 6.0%, 64.6 ± 15.3%, and 90.4 ± 5.2%, respectively) (all *p* < 0.05) and mild to moderate EDS group (43.9 ± 41.0 min, 7.9 ± 5.8%, 66.5 ± 15.1%, and 91.7 ± 4.0%, respectively) (all *p* < 0.05) were lower than those in the non-EDS group (52.6 ± 52.8 min, 9.2 ± 6.2%, 74.7 ± 11.3%, and 93.7 ± 2.6%, respectively). The SL in the severe EDS group (16.1 ± 14.4 min) was shorter than that in the mild to moderate EDS group (26.4 ± 27.7 min) (*p* < 0.05) and non-EDS group (31.0 ± 27.6 min) (*p* < 0.05). The N2 (%) in the severe EDS group (40.3 ± 16.7%) was lower than that in the non-EDS group (45.5 ± 14.2%) (*p* < 0.05). The N3% in the mild to moderate EDS group (3.1 ± 5.1%) was lower than that in the non-EDS group (4.6 ± 6.6%) (*p* < 0.05).

### 3.4. Multivariate Analysis

The results of two binary logistic regression analyses comparing the mild to moderate EDS group versus the non-EDS group and the severe EDS group versus the non-EDS group are shown in Tables 4 and 5, respectively. WC (OR = 1.060, 95% CI: 1.039 to 1.082, *p* < 0.0001), memory loss (OR = 1.859, 95% CI: 1.264 to 2.734, *p*=0.002), work/commute disturbances (OR = 1.674, 95% CI: 1.104 to 2.539, *p*=0.015, and OR = 2.395, 95% CI: 1.442 to 3.977, *p*=0.001), and sleep efficiency (OR = 1.018, 95% CI: 1.002 to 1.034, *p*=0.024) were independently associated with mild to moderate EDS, and the MAI (OR = 1.010, 95% CI: 1.001 to 1.020, *p*=0.034), AHI (OR = 1.021, 95% CI: 1.012 to 1.029, *p* < 0.0001), and SIT90 (OR = 1.010, 95% CI: 1.001 to 1.019, *p*=0.024) were independent contributing factors of mild to moderate EDS in OSA patients. Meanwhile, age (OR = 1.048, 95% CI: 1.018 to 1.079, *p*=0.001), NC (OR = 1.124, 95% CI: 1.049 to 1.205, *p*=0.001), gasping/choking (OR = 2.605, 95% CI: 1.337 to 5.078, *p*=0.005), memory loss (OR = 2.446, 95% CI: 1.091 to 5.483, *p*=0.030), work/commute disturbances (OR = 5.012, 95% CI: 2.501 to 10.046, *p* < 0.0001, and OR = 4.397, 95% CI: 2.089 to 9.257, *p* < 0.0001), and SL (OR = 0.960, 95% CI: 0.938 to 0.982, *p*=0.001) were independently associated with severe EDS, and the AHI (OR = 1.020, 95% CI: 1.002 to 1.039, *p*=0.026) and mean SpO_2_ (OR = 0.899, 95% CI: 0.812 to 0.996, *p*=0.042) were independent contributing factors of severe EDS in OSA patients.

## 4. Discussion

The present study showed that OSA patients with various severities of EDS were more obese and had more comorbid symptoms compared to patients without EDS. Multivariate analysis showed some comorbid symptoms, such as memory loss and work/commute disturbances, were independently associated with EDS and certain parameters related to sleep fragmentation, respiratory events, and nocturnal hypoxia may be predictors of EDS.

### 4.1. Demographic Characteristics

Our study showed the age of severe EDS patients was slightly greater than that of mild to moderate EDS and non-EDS patients. In contrast, Pallesen et al. showed that younger age was an independent predictor of EDS [28]. This discrepancy may be related to the study population, as Pallesen et al. enrolled adults from the general population, whereas our study enrolled patients who visited tertiary medical center for symptoms. We found that patients with EDS had greater NC, WC, and BMI. WC and NC were independently associated with mild to moderate or severe EDS, respectively. These indicated that OSA patients with EDS were more obese compared to those without EDS. Koutsourelakis et al. showed that, among patients with suspected OSA at a sleep clinic, BMI was an independent, although weak, contributing factor of subjective sleepiness [29]. Bixler et al. reached similar conclusions [30]. Although multivariate analysis did not identify BMI as an independent contributing factor of EDS in our study, univariate analysis indicated that BMI increased consistently with the aggravation of EDS. Some researchers believe that obesity and metabolic disorders may affect circadian rhythms and cause daytime sleepiness [31]. In the present study, the BMI of the study population was relatively lower than that in most of the research studies from western countries. This may reflect the distinct characteristics of most Chinese OSA patients and those who are not severely obese.

### 4.2. Clinical Manifestations and Comorbidities

We found that OSA patients with EDS were more likely to have other symptoms, such as gasping/choking at night and memory loss. However, the roles of these comorbid symptoms in the development of EDS and their clinical significance in the treatment of OSA patients are unclear. Further research is needed to answer these questions. This study showed that work/commute disturbances were more frequently accompanied by EDS, and work/commute disturbances were independently associated with EDS of various severities. Previous studies had documented daytime sleepiness as a major cause of traffic and occupational accidents [5, 6], and daytime sleepiness was considered a useful indicator to predict high-risk OSA patients prone to traffic accidents in a research. This may be attributed to the decreased psychomotor alertness in EDS patients [32]. We found patients with EDS had a high prevalence of T2DM. This interesting relationship was consistent with the previous evidence that EDS is an independent risk factor of diabetes in OSA [33] and patients with EDS are characterized by hyperglycemia and insulin resistance compared to non-EDS ones [34].

### 4.3. Polysomnographic Findings

We found that patients with EDS had increased sleep efficiency, decreased WASO, more pronounced sleep structure disturbance, more frequent respiratory events, and more severe nocturia. Multivariate analysis showed that the MAI, AHI, and SIT90 were independent contributing factors of mild to moderate EDS and the AHI and mean SpO_2_ were independent contributing factors of severe EDS. However, the odds ratios (ORs) of these PSG parameters were all modest, and thus, the clinical significance is quite limited. The insufficient sample size may be one of the reasons for low OR values. Different conclusions have been reached in previous studies regarding the independent risk factors of EDS in OSA patients. Previously, researchers believed that EDS in OSA was caused by sleep structure disorders and sleep fragmentation due to recurrent respiratory events at night [14]. Later, some researchers disagreed and proposed that EDS was related to nocturnal hypoxia rather than sleep fragmentation [15]. Moreover, some researchers found that the apnea index or AHI was the predictor of EDS [35, 36]. Another study has shown that the AHI, nocturnal hypoxia, and sleep fragmentation were all independent factors of EDS [37].

### 4.4. Possible Mechanisms of EDS

The underlying physiological mechanisms causing EDS in OSA patients remain partially unclarified. Most studies on how OSA patients develop EDS focused on the role of CIH. Animal studies have shown that CIH resulted in loss of neuronal cells associated with sleep/wake regulation, induced nitric oxide synthase release, mediated oxidative stress and inflammatory damage in the brain regions that promote wakefulness, and mediated activation of the brain regions that promote sleep, thereby leading to sleepiness [38]. Moreover, once oxidative stress is inhibited with drugs or alleviated by knocking out the NADPH oxidase gene, lipid peroxidation damage in brain regions is blocked to reduce sleepiness [39]. In addition, EDS may be related to sleepiness-associated, hypoxia-induced increases in circulatory inflammatory mediators, such as tumor necrosis factor-*α* (TNF-*α*), interleukin 6 (IL-6), and intercellular adhesion molecule-1 (ICAM-1) [40]. Vgontzas et al. showed that etanercept, a TNF-*α* receptor antagonist, significantly reduced EDS [41]. Additionally, the cyclooxygenase-2/prostaglandin D synthetase and prostaglandin in D2 signaling pathways may be involved in the development of EDS [42]. Further investigation of these mechanisms will help researchers understand the pathophysiological features of OSA and its different clinical manifestations, including EDS, and provide new directions for potential prevention strategies and treatment targets.

### 4.5. Care for Non-EDS Patients

CPAP is the first-line therapy for moderate to severe OSA, and its positive effects are more prominent in EDS patients. This is partly due to better adherence to CPAP treatment in EDS patients. However, just as shown in our study, a large number of patients do not have daytime sleepiness. The management of non-EDS subjects may be a challenge and deserves attention. Up to now, only a few studies focused on the treatment of non-EDS patients, and the results were conflicting. Large randomized clinical trials with long-term follow-up are needed to evaluate the impact of CPAP on OSA patients without EDS. On the contrary, the progress in the understanding of treatment choice for non-EDS patients is depending on more detailed identification of factors and mechanisms contributing to differentiate this distinct clinical phenotype [13].

### 4.6. Strengths and Limitations

This study has some advantages over previous studies: First, we conducted a comprehensive analysis of clinical features (including demographic information, symptoms, signs, and common comorbidities) and PSG variables (including sleep structure, respiratory events, and nocturnal hypoxia-related parameters). Meanwhile, clinical features of EDS were carefully investigated by stratifying the sample into different EDS severity groups. Second, compared with most studies in the existing literature coming from western countries, the patients included in this study had a relatively low BMI. The results of our study reflect the characteristics of Chinese patients. Moreover, we think they can also be extrapolated to other OSA populations that are not very obese. Nevertheless, this study has some limitations that should be considered when interpreting the results: First, the ESS score was used to assess subjective sleepiness. The ESS is simple and has its shortcomings. The results would be more accurate if the multiple sleep latency test (MSLT) was used to assess objective sleepiness concurrently; however, the MSLT is time-consuming and labor-intensive and is not available in our center. Second, the sample size was relatively small for such a common disease. By increasing the sample size, we may find more stronger predictors of EDS. Larger and well-controlled studies are needed in the future for more in-depth investigations.

## 5. Conclusions

The characterization of EDS is important for clinicians not only for the phenotyping of OSA but also for providing appropriate treatment. This study showed that OSA patients with EDS were more obese compared to those without EDS and had more symptoms and comorbidities such as T2DM. Multiple factors including age, NC, WC, gasping/choking, memory loss, work/commute disturbances, sleep efficiency, and SL were independently associated with EDS, and the MAI, AHI, SIT90, and mean SpO_2_ were independent contributing factors of EDS in OSA patients. We believe that when evaluating EDS and considering the diagnosis and treatment for OSA patients, clinicians should comprehensively consider demographic, clinical, and polysomnographic factors such as patients' weight, concurrent symptoms and comorbidities, sleep parameters, respiratory events, and nocturnal hypoxia rather than just focusing on the AHI.

## Figures and Tables

**Table 1:** Demographic characteristics of the total OSA patients and the patients with different severities of EDS.

	Total	Non-EDS	Mild to moderate EDS	Severe EDS	*F*/*χ* ^2^	*p*
Number of cases	874	582	227	65	—	—
Gender (male : female)	739 : 135	482 : 100	203 : 24	54 : 11	5.580	0.061
Age	45.4 ± 11.6	45.2 ± 12.0	44.5 ± 10.2	49.5 ± 11.3^a,b^	4.757	0.009
BMI (kg/m^2^)	27.2 ± 3.4	26.8 ± 3.3	27.9 ± 3.3^c^	29.1 ± 3.6^a,b^	20.462	<0.0001
NC (cm)	38.4 ± 3.8	38.1 ± 4.1	38.9 ± 3.2^c^	39.7 ± 3.4^a^	7.347	0.001
WC (cm)	97.8 ± 9.5	96.1 ± 9.0	101.0 ± 9.5^c^	102.1 ± 9.9^a^	31.003	<0.0001
Awake SpO_2_	96.6 ± 0.9	96.6 ± 0.9	96.5 ± 0.9	96.4 ± 0.8	2.338	0.097
ESS score	8.3 ± 5.0	5.4 ± 2.6	12.8 ± 1.6^c^	19.0 ± 2.0^a,b^	1499.010	<0.0001

Data are presented as the number of cases or mean ± SD. ^a^Comparisons between non-EDS versus severe EDS groups (*p* < 0.05); ^b^comparisons between mild to moderate EDS versus severe EDS groups (*p* < 0.05); ^c^comparisons between non-EDS versus mild to moderate EDS groups (*p* < 0.05). EDS: excessive daytime sleepiness; BMI: body mass index; NC: neck circumference; WC: waist circumference; ESS: Epworth Sleepiness Scale.

**Table 2:** Clinical manifestations and comorbidities of the total OSA patients and the patients with different severities of EDS.

	Total	Non-EDS	Mild to moderate EDS	Severe EDS	*χ* ^2^	*p*
Gasping/choking	38.8	33.7	46.3^c^	58.5^a^	22.331	<0.0001
Morning headache	17	12.4	23.3^c^	36.9^a,b^	33.529	<0.0001
Dry mouth	65.2	61.9	70.9^c^	75.4^a^	9.122	0.010
Increased nocturia	28	25.4	29.1	47.7^a,b^	14.530	0.001
Memory loss	61.2	55.3	70.5^c^	81.5^a^	28.023	<0.0001
Irritability	37.2	33.7	43.6^c^	46.2^a^	9.320	0.009
Life disturbance	38.8	33.0	47.1^c^	61.5^a,b^	29.075	<0.0001
Work disturbance	27.8	19.4	38.8^c^	64.6^a,b^	77.871	<0.0001
Commute disturbance	15.3	7.6	26.4^c^	46.2^a,b^	96.193	<0.0001
Hypertension	35.5	33.3	39.2	41.5	3.592	0.166
T2DM	9.3	7.0	12.3^c^	18.5^a^	12.494	0.002
COPD	4	5	1.8	3.1	4.564	0.102
IHD	9.8	9.3	9.3	16.9	3.972	0.137
Stroke	2.5	2.4	3.1	1.5	0.580	0.748
Gastroesophageal reflux	9.6	9.5	9.7	10.8	0.119	0.942
Hypothyroidism	3.3	4.0	1.8	3.1	2.454	0.293
Pharyngitis	43.6	44.5	40.5	46.2	1.236	0.539
Rhinitis	34.1	35.2	32.6	29.2	1.240	0.538
Smoking	9.4	9.6	8.8	9.2	0.128	0.938
Alcohol use	3.2	2.7	3.5	6.2	2.287	0.319
History of upper airway surgery	1.0	1.0	0.9	1.5	0.214	0.898
Mandibular retraction	5.6	5.3	7.0	3.1	1.764	0.414
Tonsil enlargement	9.2	7.2	13.2^c^	12.3	7.909	0.019
Pharyngeal stenosis	16.4	12.4	22.5^c^	30.8^a^	22.815	<0.0001

Data are presented as percentage. ^a^Comparisons between non-EDS versus severe EDS groups (*p* < 0.05); ^b^comparisons between mild to moderate EDS versus severe EDS groups (*p* < 0.05); ^c^comparisons between non-EDS versus mild to moderate EDS groups (*p* < 0.05). EDS: excessive daytime sleepiness; T2DM: type 2 diabetes mellitus; COPD: chronic obstructive pulmonary disease; IHD: ischemic heart disease.

**Table 3:** PSG variables of the total OSA patients and the patients with different severities of EDS.

	Total	Non-EDS	Mild to moderate EDS	Severe EDS	*F*	*p*
Sleep efficiency (%)	84.8 ± 12.3	83.5 ± 12.9	87.3 ± 10.3^c^	88.2 ± 10.3^a^	11.032	<0.0001
WASO (min)	49.0 ± 49.3	52.6 ± 52.8	43.9 ± 41.0^c^	34.8 ± 39.5^a^	5.537	0.004
SL (min)	28.7 ± 27.2	31.0 ± 27.6	26.4 ± 27.7	16.1 ± 14.4^a,b^	10.119	<0.0001
N1 (%)	42.7 ± 19.2	40.7 ± 17.3	46.4 ± 22.6^c^	48.5 ± 19.5^a^	10.775	<0.0001
N2 (%)	44.6 ± 14.7	45.5 ± 14.2	43.5 ± 15.3	40.3 ± 16.7^a^	4.405	0.012
N3 (%)	4.2 ± 6.4	4.6 ± 6.6	3.1 ± 5.1^c^	4.6 ± 7.3	4.867	0.008
REM (%)	8.7 ± 6.2	9.2 ± 6.2	7.9 ± 5.8^c^	6.6 ± 6.0^a^	7.922	<0.0001
MAI (h^−1^)	44.4 ± 20.6	40.8 ± 19.4	51.5 ± 20.7^c^	51.4 ± 22.9^a^	27.643	<0.0001
AHI (h^−1^)	46.7 ± 24.7	36.6 ± 23.2	54.4 ± 22.8^c^	55.7 ± 24.7^a^	58.831	<0.0001
TAmax (s)	60.2 ± 27.3	56.2 ± 25.7	68.7 ± 28.7^c^	66.1 ± 28.5^a^	19.781	<0.0001
Nadir SpO_2_	71.8 ± 13.4	74.7 ± 11.3	66.5 ± 15.1^c^	64.6 ± 15.3^a^	45.422	<0.0001
Mean SpO_2_	92.9 ± 3.4	93.7 ± 2.6	91.7 ± 4.0^c^	90.4 ± 5.2^a^	50.413	<0.0001
ODI (h^−1^)	43.1 ± 25.8	36.9 ± 24.1	54.8 ± 24.3^c^	57.2 ± 26.2^a^	55.829	<0.0001
SIT90 (%)	16.0 ± 22.9	12.3 ± 16.6	22.6 ± 32.4^c^	26.5 ± 23.5^a^	25.205	<0.0001

Data are presented as mean ± SD. ^a^Comparisons between non-EDS versus severe EDS groups (*p* < 0.05); ^b^comparisons between mild to moderate EDS versus severe EDS groups (*p* < 0.05); ^c^comparisons between non-EDS versus mild to moderate EDS groups (*p* < 0.05). EDS: excessive daytime sleepiness; WASO: wake after sleep onset; SL: sleep latency; REM: rapid eye movement; MAI: microarousal index; AHI: apnea-hypopnea index; TAmax: the longest time of apnea; ODI: oxygen desaturation index; SIT90: saturation impair time below 90%.

**Table 4:** Multivariate analysis of mild to moderate EDS by binary logistic regression.

	*B*	SE	Sig.	OR	95% CI
WC	0.058	0.010	<0.0001	1.060	1.039–1.082
Memory loss	0.620	0.197	0.002	1.859	1.264–2.734
Work disturbance	0.515	0.212	0.015	1.674	1.104–2.539
Commute disturbance	0.873	0.259	0.001	2.395	1.442–3.977
Sleep efficiency	0.018	0.008	0.024	1.018	1.002–1.034
MAI	0.010	0.005	0.034	1.010	1.001–1.020
AHI	0.020	0.004	<0.0001	1.021	1.012–1.029
SIT90	0.010	0.004	0.024	1.010	1.001–1.019
Constant	−11.115	1.299	<0.0001	0.000	—

For categorical variables, 0 indicates “female or no” and 1 indicates “male or yes.” Only statistically significant variables (*p* < 0.05) are shown. WC: waist circumference; MAI: microarousal index; AHI: apnea-hypopnea index; SIT90: saturation impair time below 90%; *B*: partial regression coefficient; SE: standard error; OR: odds ratio; CI: confidence interval.

**Table 5:** Multivariate analysis of severe EDS by binary logistic regression.

	*B*	SE	Sig.	OR	95% CI
Age	0.047	0.015	0.001	1.048	1.018–1.079
NC	0.117	0.035	0.001	1.124	1.049–1.205
Gasping/choking	0.958	0.340	0.005	2.605	1.337–5.078
Memory loss	0.894	0.412	0.030	2.446	1.091–5.483
Work disturbance	1.612	0.355	<0.0001	5.012	2.501–10.046
Commute disturbance	1.481	0.380	<0.0001	4.397	2.089–9.257
SL	−0.041	0.012	0.001	0.960	0.938–0.982
AHI	0.020	0.009	0.026	1.020	1.002–1.039
Mean SpO_2_	−0.106	0.052	0.042	0.899	0.812–0.996
Constant	−1.300	5.487	0.813	0.272	—

For categorical variables, 0 indicates “female or no” and 1 indicates “male or yes.” Only statistically significant variables (*p* < 0.05) are shown. NC: neck circumference; SL: sleep latency; AHI: apnea-hypopnea index; *B*: partial regression coefficient; SE: standard error; OR: odds ratio; CI: confidence interval.

## Data Availability

The SPSS data used to support the findings of this study are available from the corresponding author upon request.

## Abbreviations

AASM: American Academy of Sleep Medicine
AHI: Apnea-hypopnea index
BMI: Body mass index
CIH: Chronic intermittent hypoxia
COPD: Chronic obstructive pulmonary disease
CPAP: Continuous positive airway pressure
ECG: Electrocardiogram
EDS: Excessive daytime sleepiness
EEG: Electroencephalogram
EMG: Electromyogram
EOG: Electrooculogram
ESS: Epworth Sleepiness Scale
ICAM-1: Intercellular adhesion molecule-1
ICSD: International Classification of Sleep Disorders
IL-6: Interleukin 6
ODI: Oxygen desaturation index
OHS: Obesity hypoventilation syndrome
OR: Odds ratio
OSA: Obstructive sleep apnea
MSLT: Multiple sleep latency test
SIT90: Saturation impair time below 90%
MAI: Microarousal index
NC: Neck circumference
PSG: Polysomnography
TAmax: The longest time of sleep apnea
T2DM: Type 2 diabetes mellitus
NADPH: Nicotinamide adenine dinucleotide phosphate
SL: Sleep latency
TNF-*α*: Tumor necrosis factor-*α*
TST: Total sleep time
WASO: Wake after sleep onset
WC: Waist circumference.
